# “Underground innovation” in the digital age: how digital leadership promotes employee bootlegging through psychological empowerment

**DOI:** 10.3389/fpsyg.2026.1914919

**Published:** 2026-08-17

**Authors:** Lulu Song, Yixuan Chen, Zhen Wang

**Affiliations:** Zhejiang University of Finance & Economics Dongfang College, Haining, China

**Keywords:** digital leadership, employee bootlegging, organizational formalization, psychological empowerment, self-determination theory

## Abstract

**Background:**

Under the trend of digitalization, the employees’ “informal” innovative behaviors are of great significance for enterprises to adapt to highly uncertain market environments.

**Methods:**

Based on Self-Determination Theory, this study builds a moderated mediation model to examine how digital leadership promotes employee bootlegging through psychological empowerment, and also considers the moderating role of organizational formalization in this path.

**Results:**

Through an empirical analysis of 387 valid survey questionnaires from employees in multi-regional enterprises, and on the basis of the above research results: (1) Digital leadership is positively associated with the psychological empowerment of employees; (2) Psychological empowerment mediates the impact of digital leadership on employees’ bootlegging behavior; (3) Organizational formalization strengthens the path from psychological empowerment to bootlegging, meaning that a more complex procedural system at the company increases the risk of such behavior among employees with high psychological empowerment; (4) Organizational formalization further positively moderates the indirect path of “digital leadership—psychological empowerment—employee bootlegging.”

**Conclusion:**

This study reveals the micro-psychological mechanisms of employees under the conflict between digital empowerment and bureaucratic systems, providing important theoretical perspectives and managerial insights for organizations to optimize innovation management and reshape institutional environments in the digital era.

## 1 Introduction

With the development of artificial intelligence and the digital economy, many new uncertainties and problems have arisen in the lives and development of enterprises today. To be competitive in the highly competitive market, more and more enterprises have been promoting digitalization by using bottom-up employee innovation (Nambisan et al., 2017). Digitalization requires the introduction of new tools and is also needed to reform the old management system fundamentally (Kane et al., 2015). Therefore, digital leadership has appeared as a new model for leadership. In recent years, with the rapid advancement of digital transformation research, scholars have increasingly recognized that digital leadership has evolved from a mere technological advocacy role into a strategic organizational capability that influences innovation performance, sustainable development, and employee behavior (Khan et al., 2024; Huang et al., 2026). Digital Leaders can provide numerous digital resources, cultivate an agile and iterative culture, foster open collaboration, etc.; therefore, they are one of the main reasons that motivate employees to adapt to changes in digital technology and fully realize their innovative capacity (Zhu et al., 2022; Mboungou, 2024). Empirical research further indicates that digital leadership not only significantly enhances employees’ innovative work behavior (Abbas et al., 2024; Hang and Nguyet, 2025) but also drives employees’ green innovation behavior by shaping a green organizational climate (Khan et al., 2025), and even promotes the synergistic development of exploitative and exploratory innovation at the organizational level (Ying et al., 2026). However, most organizational behavior studies have assumed that a good leader will directly motivate employees to engage in formally approved and organizationally sanctioned innovation; they have not considered that in complex organizations, employees’ desire to innovate may take the form of “informal” behavior or even “deviant” behavior.

Employee bootlegging is an extraordinary type of innovation that has attracted the attention of scholars all over the world recently. It refers to the situation where employees invest their time and resources in exploratory research for new applications of their work in their spare time and on their own initiative, without seeking official approval from their superiors or following established approval procedures. Traditional management views “deviance” or “rule-breaking” as failures in maintaining the order of the organization. However, many studies on innovation governance have shown that during times of rapid change in the digital era, many groundbreaking technologies and important achievements have originated from focused anticipatory informal probing (Howell and Higgins, 1990). Digital leadership has a significant positive impact on employee bootlegging, with employee ambition playing an important mediating role in this relationship (Sun et al., 2024). A problem of theories arises: If organizations are now giving more power to digital leaders, why are employees still resorting to underground “bootlegging” for income at the risk of their careers? Existing literature has not been able to explain why the micro-psychological black box and the boundaries of the context have not been studied.

To address the above shortcomings in theory, Self-Determination Theory (SDT) will be applied in this paper, and psychological empowerment will serve as the mediator in the research. Based on the theoretical framework of SDT, the latest research indicates that digital leadership can drive innovative behavior by stimulating employees’ intrinsic motivation. Digital leaders satisfy employees’ basic psychological needs for autonomy, competence, and relatedness by providing autonomy support and capacity building, thereby igniting and sustaining employees’ intrinsic motivation for innovation (Huang et al., 2026). Digital leadership provides technological support for the deep-seated mental demands of modern employees for autonomy and professional self-realization through empowerment and flatter organizations; as a result, it can foster strong affective commitment and intrinsic motivation (Li et al., 2025). Whenever employees believe they have a relatively high degree of empowerment and self- determination in their work, they are no longer bound by duty but are more likely to be motivated by a sense of purpose and an eagerness to learn and grow (Zhang and Bartol, 2010). This relatively high level of intrinsic motivation, which is driven by a sense of empowerment, is widely considered to be the primary motivation for people to go beyond their jobs and take on high-risk proactive behaviors (Parker et al., 2010). Recently, based on psychological studies, people in a time of change and uncertainty have been able to generate creative ideas while maintaining good mental health (Bai et al., 2023).

However, the change in intrinsic motivation to bootlegging behavior does not happen in isolation; it is inevitably limited by the internal institutional environment of the organization. Therefore, this study will also add organizational formalization to the model. Based on the above studies, organizational pressure and a rigid bureaucratic system may have lowered people’s psychological energy and led them to adopt a defence-oriented coping mode (Wu and Gao, 2025). According to the findings in organizational behavior, a number of factors limit creativity in work today (Hirst et al., 2011). It is noteworthy that while digital leadership drives innovation, its effectiveness is highly dependent on the alignment of the organizational institutional environment. When enterprises face significant operational difficulties, the governance effect of digital leadership in alleviating innovation information constraints becomes more pronounced (Li and Tian, 2025), indicating that the impact of digital leadership is profoundly shaped by the organizational context. Highly psychologically empowered employees are in a rigid organizational setting that is overly formalized, has strict rules and lengthy approval procedures, and thus find themselves at odds with their strong internal drive for innovation. To prevent the loss of inspiring ideas due to multiple rejections, empowered staff have had to quietly turn away from formal channels for innovative work. On the other hand, if the organization has a flexible and error-tolerant environment (Huang et al., 2022), then “underground operations” are not required to this extent.

Although the above theoretical deduction preliminarily reveals the potential connections among digital leadership, psychological empowerment, and the organizational institutional environment, three research gaps in the existing literature urgently need to be addressed. First, at the mechanism level, existing studies have mostly focused on the linear impact of digital leadership on formal innovation performance or compliance behavior (Khan et al., 2024; Abbas et al., 2024), yet few have opened the micro-psychological black box of how it induces “informal” bootlegging. Whether and how psychological empowerment plays an irreplaceable role as a “motivational converter” between digital leadership and bootlegging remains lacking direct empirical testing. Second, at the contextual boundary level, the role of organizational formalization in innovation management has long been subject to a theoretical paradox. While some studies emphasize its negative side of stifling flexibility (Adler and Borys, 1996), others point to its positive side of providing stable expectations. However, in the specific context of digital empowerment, does rigid bureaucracy suppress employees’ innovative impulses, or does it instead “squeeze” the creativity of highly psychologically empowered employees into an underground state? This paradox has yet to be clearly resolved in the existing literature on bootlegging (Globocnik and Salomo, 2015). Third, at the theoretical integration level, although self-determination theory is widely used to explain the formation of intrinsic motivation, few studies have placed it within the tension field of “digital empowerment” and “bureaucratic constraints” to systematically examine the complex interaction mechanisms among the three in stimulating employee bootlegging. In light of this, this study aims to construct a moderated mediation model to systematically address the above gaps from the perspective of SDT theory.

In short, this study presents a moderated mediation model to examine the path by which digital leadership affects employee bootlegging behaviors through psychological empowerment and identifies the moderating role of organizational formalization on this change in psychology. The expected contributions of this work are as follows: First, it extends the scope of outcomes for digital leadership by moving away from the traditional view that “positive leadership will necessarily result in formal compliant behavior”; Second, it adds new contents to the antecedent research on bootlegging, providing support from the perspective of SDT theory to suggest that the core of bootlegging may be the informal release of “suppressed intrinsic motivation”; Third, it offers a warning to corporate management practices in the era of digital transformation that simply providing digital empowerment is not enough. If the rigid bureaucratic system is not reformed at the same time, high-innovation enthusiasm will only flow into unregulated “underground organizations.”

## 2 Theoretical basis and research hypotheses

### 2.1 Overall theoretical framework: self-determination theory

Deci and Ryan developed Self-Determination Theory, and it serves as the theoretical basis for this study. SDT is an all-encompassing meta-theory of people’s motivations and growth, and it is not restricted to external stimulus reinforcement. It is believed that individual behavior is motivated by the three basic psychological needs for autonomy, competence and relatedness (Deci, 2000). After meeting the above basic psychological needs, people will have intrinsic motivation to act autonomously and are likely to be proactive, creative and flexible (Gagné and Deci, 2005).

In recent years, scholars have increasingly studied how Self-Determination Theory is applied to the workplace to understand why some employees are more motivated and creative at work (Deci et al., 2017). In the digital work environment, Self-Determination Theory has been proven to be an effective framework for understanding the generation mechanism of employees’ innovative behavior. The sequential mediation model constructed by Huang et al. (2026) based on Self-Determination Theory indicates that digital leadership drives innovative behavior by stimulating employees’ intrinsic motivation. Digital leaders satisfy employees’ basic psychological needs for autonomy, competence, and relatedness by providing autonomy support and capacity building, thereby igniting and sustaining employees’ intrinsic innovation motivation. Similarly, Wang Y. et al. (2025) found that digital leadership significantly enhances employees’ innovation performance by promoting job crafting. Furthermore, research by Li et al. (2024) based on social exchange theory also confirmed that middle managers’ digital leadership significantly strengthens employees’ perception of empowerment, thereby increasing work engagement. Given the change in the corporate environment due to the digital transformation process, the scale and unpredictability of innovation at present have been increasing substantially (Amabile, 1996). According to the internal logical chain of SDT theory, which is “environmental support → psychological need satisfaction → behavioral output,” this study builds a new theoretical model: digital leadership is viewed as the “environmental antecedent” that offers autonomy support and resource empowerment in the organization; psychological empowerment is regarded as the core “internal motivational state” resulting from the fulfilment of individuals’ basic psychological needs; employee bootlegging behavior is considered the finally activated “proactive behavioral script”; and organizational formalization is treated as the “external situational constraint” that individuals face in implementing innovation. Through an in-depth study of the network connections and sequential activation mechanisms among these variables, this paper seeks to explore the psychological “black box” and macro-institutional boundaries for employee deviant innovative behavior in the digital age.

### 2.2 The relationship between digital leadership and psychological empowerment

Within the framework of SDT theory, leaders’ management behaviors are the most important contextual cues in an individual’s work environment, directly determining the degree to which their psychological needs are satisfied (Gagné and Deci, 2005). Digital leadership refers to the leadership behaviors in which leaders advocate for and promote the application of digital technology within an organization, leading the team through digital transformation with an agile and open mindset (Zeike et al., 2019). In the face of a rapidly evolving digital business environment, managers with high digital leadership not only provide tools but also foster an empowering work atmosphere.

Based on early motivational models, scholars have proposed that psychological empowerment is a comprehensive internal motivational state composed of four cognitive dimensions (meaning, competence, autonomy, and impact) (Thomas and Velthouse, 1990; Spreitzer, 1995). The empowerment and activation of employees’ psychological empowerment by digital leadership are reflected in the following three psychological processing levels: First, digital leaders actively introduce AI and cutting-edge digital tools and encourage employees to learn through trial and error. This resource support and skill development greatly satisfy employees’ “competence,” making them confident in their ability to complete complex tasks (Erhan et al., 2022). Li et al. (2024) confirmed that higher levels of digital leadership are significantly associated with employees’ stronger perception of empowerment; Zhang et al. (2026b), using a dual-method approach (PLS-SEM and fsQCA), found that digital leadership significantly influences employees’ flow experience and improvisational ability through the key moderating variable of psychological safety. Flow, as a deep intrinsic motivational state, is precisely one of the core manifestations of psychological empowerment, providing cutting-edge empirical support for the activation of psychological empowerment by digital leadership. Second, in the leadership paradigm of the digital era, managers increasingly advocate for decentralization and agile work methods, granting subordinates a high degree of freedom (Bennis and Nanus, 1985). Thus, such a delegation of authority fulfills the employee’s desire for “self-determination” and gives them a strong sense of responsibility for their work. Digital leaders can stimulate employees’ innovation performance by shaping a digital culture, allocating key resources, and optimizing organizational processes Wang Y. et al., 2025). Finally, the digital transformation vision articulated by leaders helps employees connect their daily work to the organization’s broader goals, thereby significantly enhancing their perception of “work meaning.” Based on the systematic deduction from the SDT theoretical perspective, this study proposes the following hypothesis:

*H1*: Digital leadership has a significant positive impact on employees’ psychological empowerment.

### 2.3 The relationship between psychological empowerment and employee bootlegging

Employee bootlegging is a very specific type of innovation behavior that refers to employees privately and covertly conceiving and validating innovative ideas for the potential benefit of the organization without formal authorization from their superiors or within the normal course of work (Augsdorfer, 2005; Criscuolo et al., 2014). According to the development of SDT theory, the intrinsic motivation of people refers to those motivated by their own interest in an activity. Psychological empowerment is a very high level of internal drive that explicitly determines whether individuals are willing to assume reputational and professional risks and create non-conventional behavioral blueprints in highly ambiguous innovative environments (Seibert et al., 2011). Zhao et al. (2025) pointed out that bootlegging is prevalent among the new generation of employees, and employees’ perception of organizational error tolerance culture significantly and positively predicts bootlegging behavior. Zhang et al. (2025) further found that psychological well-being and psychological entitlement play a significant serial mediating role between leadership and employee bootlegging.

Innovation is about changing the old way of doing things; therefore, it is often not approved at the top level at the start (March, 1991). Psychological empowerment offers the employees’ primary psychological motivation to face the risks of “underground innovation.” Theoretically, employees with higher levels of psychological empowerment are more likely to view bootlegging as a form of “positive deviance,” seeking breakthroughs by challenging bureaucratic norms (Wang Q. et al., 2025). First; that is, individuals with a higher degree of psychological empowerment are more likely to possess a strong sense of self-efficacy (competence) and role tolerance (autonomy). They are not limited to a single position; they believe they can both perform the duties of their own positions and lead the change of technology or production processes in the company (Mainemelis, 2010). Second, bootlegging is an “underground state” that lacks formal resource support and is thus highly vulnerable to setbacks (Koch and Leitner, 2008). However, employees with a high sense of psychological empowerment have experienced a profound sense of purpose in their work, are more resilient in terms of psychological capital, and can still pursue their own interests and explore out of a desire for self-realization. Given that the active-behavioral nature of SDT, employees with a high level of psychological empowerment are no longer passive carriers of the company’s policies. A stronger sense of self-discipline will help them reduce their dependence on the system and build something new with their own money. Authentic leadership has a significant positive impact on employee bootlegging behavior (Liu et al., 2025). Based on this, the following hypothesis is proposed:

*H2*: Psychological empowerment has a significant positive impact on employee bootlegging behavior.

### 2.4 The mediating role of psychological empowerment

Based on the theoretical logic of H1 and H2, this study provides an all-encompassing transmission path for “environmental support - motivation arousal - behavior output” under the umbrella of SDT theory. Although digital leadership is a necessary enabling condition of the environment for providing employees with digital tools and an open-door policy, external support from the environment cannot directly and automatically translate into the behavior of taking considerable risks by employees to “deviate.”

Based on the intersection of organizational empowerment theory and Self-Determination Theory (SDT), external structural empowerment needs to be fully internalized by individuals to activate their inner “motivational cognitive hub” and achieve a leap in complex behavior scripts (Conger and Kanungo, 1988; Deci, 2000). Psychological empowerment has done some good work. On the one hand, digital leadership empowers people’s psychological capital by granting them more autonomy and training them in new-age skills to feel more in control of their work. At the same time, strong intrinsic motivation (Gagné and Deci, 2005) will also be directed at behavior; therefore, when employees do not feel that innovations are required, they may be less eager to take risks and experiment informally. That is to say, by promoting the deep processing of “motivation internalization” (thus strengthening employees’ psychological empowerment), digital leadership ultimately leads to the intrinsic stimulation of bootlegging behavior. Meanwhile, Dong et al. (2026) found that digital leadership significantly enhances employees’ taking charge behavior by promoting job crafting; Zhang et al. (2026a), from the perspective of responsible leadership, confirmed the sequential mediating role of psychological mechanisms and knowledge search between leadership and sustainable performance. This directly echoes the transmission chain of “environmental antecedents → psychological empowerment → behavioral output” in this study, providing strong theoretical evidence for psychological empowerment as a mediating hub. Based on this, the following hypothesis is proposed:

*H3*: Psychological empowerment plays a significant mediating role between digital leadership and employee bootlegging.

### 2.5 The moderating effect of organizational formalization and the moderated mediation mechanism

According to the theory of self-determination, the environment will substantially impact how people exhibit their own will-as-reason for behavior. Therefore, this paper will take organizational formalization as a moderator. Formalization refers to the extent of complexity and rigidity in the rules, procedures and approval processes of an organization (Jaworski and Kohli, 1993). Though the system of the process provides certain expectations for all involved, if it is too rigid, an inflexible “coercive bureaucratic system” will result (Adler and Borys, 1996).

This study posits that organizational formalization is an external institutional context that enhances and motivates a transformation from psychological empowerment into bootlegging behavior, as shown in this study. In short, the empowered employees are more motivated to innovate out of a sense of purpose. However, when the degree of organizational formalization is too high (prolonged approval procedures and inflexible rules), employees’ motivation for innovation is severely suppressed. If they submit their new ideas through official channels, they are likely to be rejected at various stages in the multiple-layered approval process and thus miss short-lived market opportunities (Globocnik and Salomo, 2015). To protect and promote their good ideas for innovation, employees who are highly motivated internally may feel that they have “no choice” but to turn their innovation efforts into unofficial, underground ones, thus increasing the incentive for bootlegging (Mainemelis, 2010). On the other hand, in the absence of strong organizational formalization and a flatter communication structure (Mintberg, 1989), employees with high psychological empowerment can more easily obtain top-down support through regular channels; as a result, the demand for “underground operations” will decline, and their incentive to be creative may be more readily realized through official channels rather than illegal means. Therefore, the following hypothesis is proposed:

*H4*: Organizational formalization increases the positive effect of psychological empowerment on employee bootlegging. Organizational formalization will likely strengthen the reinforcing effect of psychological empowerment on employee bootlegging behavior.

Furthermore, from the global dynamic perspective of the theoretical model, this moderating effect will propagate upward along the entire mediation chain. Since a high level of organizational formalization reinforces the inevitability of psychological empowerment being forced to transform into bootlegging at the end, it will inevitably amplify the entire indirect pathway through which digital leadership induces bootlegging via psychological empowerment. Specifically, when organizational systems are highly rigid, the psychological empowerment granted by digital leaders to employees resembles a “suppressed volcano,” ultimately erupting only through informal “deviant” fissures; in contrast, in organizations with flexible systems, this indirect pathway has a relatively weaker effect on promoting bootlegging. Accordingly, a moderated mediation hypothesis is proposed:

*H5*: Organizational formalization positively moderates the mediating role of psychological empowerment between digital leadership and employee bootlegging. That is, in a highly formalized organizational context, the indirect positive effect of this mediating path will be significantly enhanced.

**Figure 1:**
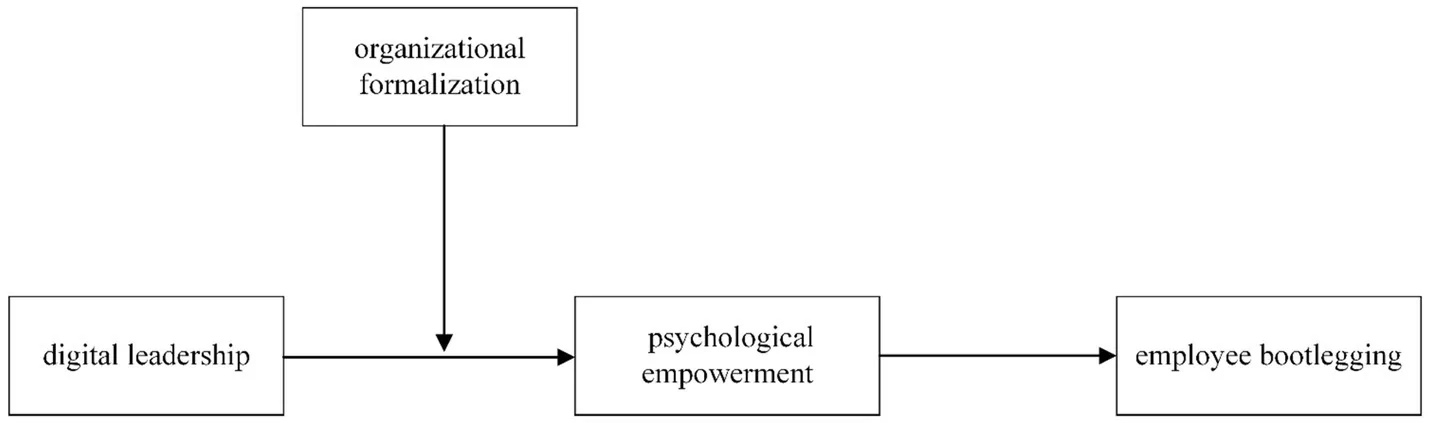
Research conceptual model.

In summary, the conceptual model of this study is shown in Figure 1.

## 3 Research design

### 3.1 Variable measurement

This study adopted established measurement scales, with all items scored on a 7-point Likert scale (1 = strongly disagree, 7 = strongly agree).

(1) *Digital leadership*: Measurement adapted from the scale by Zeike et al. (2019) and combined with the enterprise digital context of this study is carried out through six items: “My leader finds the use of digital tools interesting,” “My leader is an expert in the field of digital technology,” “My leader keeps learning about changes in digital technology,” “My leader actively leads the push for digitalization in our department,” “My leader can inspire others about digital transformation,” and “My leader promotes the application of digital tools within the team.”
(2) *Psychological empowerment*: According to Spreitzer’s (1995) original scale, the four levels of this dimension are meaning, competence, autonomy and impact. Given the length of the full scale, only representative items were selected for this study, which include: “The work I do is very important to me,” “I am confident in my ability to complete my work,” “I have a large degree of freedom in how I do my work,” and “I have significant power to affect what happens in my department,” a total of 12 items were selected for measurement.
(3) *Organizational formalization*: Based on Jaworski and Kohli’s (1993) seven items, seven indicators of the measurement were developed. The first five were reverse-scored as follows: “In most cases, I feel that I can manage by myself (reverse coded),” “Here, people are allowed to make their own decisions without asking others (reverse coded),” “Employees are frequently checked for violations of rules,” and “Here, everyone feels that they are always under surveillance to ensure adherence to rules and regulations.”
(4) *Employee bootlegging*: Measurement draws on the classic scale by Criscuolo et al. (2014), starting from the characteristics of concealment and informal exploration. The five items are: “I have worked on developing new ideas without formal approval from my superiors,” “I have informally advanced new ideas in my work,” “I have informally organized resources to test a new idea,” “I actively spend time on informal projects to lay the groundwork for future formal projects,” and “Beyond my main job responsibilities, I enjoy thinking about new ideas.” The details are shown in Table 1.

**Table 1:** Variable measurement.

Variable type	Variable name	Number of items	Scale source
Independent variable	Digital leadership	6	Zeike et al. (2019)
Mediating variable	Psychological empowerment	12	Spreitzer (1995)
Moderating variable	Organizational formalization	7	Jaworski and Kohli (1993)
Dependent variable	Employee bootlegging	5	Criscuolo et al. (2014)

### 3.2 Sample data statistics

A questionnaire survey was conducted in this study from May 2025 to January 2026, and the subjects of the survey were mainly working people of different enterprises across many regions of the country. In terms of sampling strategy, this study combines convenience sampling with snowball sampling. It distributes questionnaire links to multiple enterprises across various regions nationwide through their human resources departments, while also leveraging the social networks of current employees for moderate dissemination. A large number of questionnaires were collected in an online survey. The survey homepage explicitly informed respondents of the academic purpose, the assurance of anonymity, and the principle that the data would be used solely for statistical analysis. All respondents voluntarily participated after providing informed consent. Invalid samples were excluded due to incomplete responses or duplicate IP addresses, and 387 valid questionnaire samples were finally obtained. The actual response rate meets the requirements for social science research, and the descriptive statistics are as follows.

In terms of gender distribution, males account for 52.0% and females 48.0%, with a generally balanced ratio that effectively avoids gender bias interfering with the research conclusions. In terms of age distribution, the sample covers all age groups, with the 31–40 years age group having the highest proportion (32.0%). The overall sample is predominantly composed of young and middle-aged employees, which aligns with the characteristics of the mainstream workforce in the current context of digital transformation. In terms of education level, the largest proportion holds a bachelor’s degree (48.0%), while the sample also covers multiple education levels from high school to master’s degree, enabling the reflection of employees’ perceptions of digital leadership across different educational backgrounds. In terms of enterprise type, the sample includes state-owned, private, foreign-funded, and public institutions, with private enterprises accounting for the highest proportion (38.0%), which is consistent with the distribution characteristics of enterprise types that are more active in digital transformation practices. In terms of work experience, the sample covers different career stages, with the 1–3 years group having the highest proportion (25.0%). The overall distribution is relatively even, allowing for the reflection of differences in work experiences among employees with varying levels of seniority. In terms of job category, R&D/technical positions account for the highest proportion (28.0%), and the sample covers a wide range of job Category, including R&D technology, marketing and sales, production operations, and administrative functions, ensuring good coverage across job categories.

In short, the survey sample of this study has a relatively even distribution across various dimensions, including but not limited to gender, age, education level, enterprise type, work experience and job category. It can effectively reveal the different features of employees from various backgrounds and provide a suitable sample basis for the following research on the mechanism of digital leadership and employee bootlegging. The sample is typical of the whole and serves as an example, as shown in Table 2.

**Table 2:** Descriptive statistics of the survey sample.

Basic classification	Option	Frequency	Relative frequency (%)
Gender	Man	201	52.0%
	Female	186	48.0%
Age	25 years old and under	70	18.0%
	26–30 years old	108	28.0%
	31–40 years old	124	32.0%
	41–50 years old	62	16.0%
	51 years old and above	23	6.0%
Education level	High school/ technical secondary school and below	31	8.0%
	Junior college	85	22.0%
	Bachelor’s degree	186	48.0%
	Master’s degree and above	85	22.0%
Enterprise type	State-owned enterprises	101	26.0%
	Private enterprises	147	38.0%
	Foreign-funded / Sino-foreign joint ventures	81	21.0%
	Public institutions / Non-profit organizations	39	10.0%
	Others	19	5.0%
Work experience	Less than 1 year	46	12.0%
	1–3 years	97	25.0%
	4–5 years	89	23.0%
	6–10 years	97	25.0%
	More than 10 years	58	15.0%
Job category	R&D / Technical positions	108	28.0%
	Marketing / Sales positions	85	22.0%
	Production / Operations positions	70	18.0%
	Administrative / Functional positions	85	22.0%
	Others	39	10.0%

## 4 Data analysis and results

To ensure research reliability and the validity of related inferences, this study utilized SPSS 27.0 to control for common method bias, test scale reliability, and conduct basic variable analysis. Amos 28.0 was employed to test scale validity and model fit. The core testing procedures included three modules: common method bias testing, reliability analysis, and validity analysis, thereby eliminating random errors and systematic biases in the data, providing a solid and reliable data foundation for subsequent moderated mediation effect testing. Finally, the moderated mediation effect was tested using the PROCESS macro with the Bootstrap method.

### 4.1 Selection of analysis method

This study employs Structural Equation Modeling (SEM) combined with the PROCESS macro as the core analytical framework, a choice grounded in the following methodological considerations.

First, the advantages of SEM in handling complex mediation models. This study constructs a moderated mediation model that includes both mediation and moderation pathways, involving a complex network of relationships among multiple latent variables and their observed indicators. Compared to traditional regression analysis or path analysis, SEM offers the following irreplaceable advantages: (1) Measurement error control. SEM can separate measurement error from the estimation of latent variables, thereby obtaining more precise structural parameter estimates, which is particularly important for organizational behavior research that relies on self-report scales; (2) Overall model fit assessment. SEM provides multiple fit indices that can evaluate the consistency between the theoretical model and observed data at a global level, rather than testing the significance of individual paths in isolation; (3) Simultaneous estimation of multiple pathways. SEM can estimate direct effects, indirect effects, and moderation effects simultaneously within a single analytical framework, avoiding the accumulation of Type I error risk caused by batch testing in multiple regression analysis. Given that the theoretical model of this study involves sequential activation and interactive influences among multiple variables, the above advantages make SEM the most suitable analytical tool.

Second, the specific advantages of the PROCESS macro in testing moderated mediation. Although SEM provides an overall model fit assessment, the PROCESS macro offers a more direct and flexible testing approach for the precise quantification of moderated mediation: (1) Bootstrap confidence interval method. PROCESS directly estimates the sampling distribution of the indirect effect through repeated sampling and provides bias-corrected bootstrap confidence intervals. This method does not rely on the traditional normal distribution assumption and has higher testing power in small samples or non-normal distribution scenarios; (2) Point estimation of conditional indirect effects. PROCESS can output conditional indirect effects and their confidence intervals at different levels of the moderator variable, allowing researchers to intuitively assess the direction and strength of the moderation effect under different conditions; (3) Index of Moderated Mediation. Proposed by Hayes (2015), this index quantifies the impact of the moderator variable on the entire mediation pathway with a single statistic and directly tests its significance through bootstrap confidence intervals, providing the most direct statistical evidence for the moderated mediation hypothesis. Based on the above reasons, after completing the overall model fit assessment with SEM, this study further employs the PROCESS macro to precisely test the moderated mediation effect.

Third, the complementary use strategy of SEM and PROCESS. The analytical strategy of this study is not a simple substitution but rather fully leverages the respective advantages of the two analytical tools. First, SEM is used to conduct confirmatory factor analysis (CFA) of the measurement model and path testing of the structural model, confirming the measurement validity of theoretical constructs and the overall model fit. Second, PROCESS is employed for Bootstrap resampling to precisely quantify indirect effects and their significance under different contexts. This two-stage analytical strategy of “overall assessment—precise testing” has been widely adopted in recent methodological practices in organizational behavior and psychological research, effectively balancing the systematic nature of model fit with the precision of hypothesis testing.

### 4.2 Common method Bias test

Harman’s single-factor test is used to reduce the problem of common method bias. All the scale items were subjected to Exploratory Factor Analysis (EFA) with unrotated principal components. Based on the above tests, the explained variance of the primary factor was only 29.5% and thus did not meet the required minimum of 30%; therefore, no significant common-method bias was found in this study. Therefore, the potential for common-source bias in the survey data set will not significantly affect the final research results.

### 4.3 Reliability analysis

Since all core variables in this study are measured using established scales, assessing the reliability of the measurement tools is a key prerequisite for ensuring the validity of subsequent empirical analysis. This study adopts Cronbach’s *α* coefficient, a widely accepted indicator in academia, as the primary measure of reliability. The general criteria are as follows: An α coefficient greater than 0.8 is considered good internal consistency reliability; a coefficient between 0.7 and 0.8 is deemed acceptable reliability; and a coefficient below 0.7 indicates that the scale items need to be revised or excluded.

The reliability test results of this study are shown in Table 3. The Cronbach’s α coefficients for the four core variables, namely digital leadership, psychological empowerment, organizational formalization, and employee bootlegging, are 0.901, 0.956, 0.919, and 0.871, respectively. All α coefficients exceed the good reliability threshold of 0.8. This indicates that all scales used in this study possess excellent internal consistency and structural stability, providing stable and accurate quantitative data support for the study of the relationship between digital leadership and employee bootlegging.

**Table 3:** Reliability analysis of the questionnaire.

Variable	Cronbach’s α	Number of projects
Digital leadership	0.901	6
Psychological empowerment	0.956	12
Organizational formalization	0.919	7
Employee bootlegging	0.871	5

### 4.4 Exploratory factor analysis

First, the KMO measure and Bartlett’s test of sphericity were employed to determine whether the empirical data satisfied the conditions for factor analysis. The results show that the KMO value is 0.953; it is above 0.8 and thus considered highly reliable. In addition, the approximate chi-square statistic for Bartlett’s test of sphericity was 8181.652, and the significance level (Sig) reached 0.000, which is less than 0.001; therefore, the data are well-aggregated and meet the necessary conditions for factor analysis. As a result, principal component analysis with varimax rotation was used, and the four factors that collectively accounted for 62.838% of the variance were extracted. All the items had loadings greater than 0.50 on their corresponding factors, and there were no significant cross-loadings; thus, a good scale had been constructed.

### 4.5 Confirmatory factor analysis

The first validation of the Exploratory Factor Analysis was conducted, and then Confirmatory Factor Analysis (CFA) was carried out in this study. Structural Equation Models were used to determine whether the measurement model proposed by this paper could accurately reproduce the sample observation data. AMOS 28.0 data analysis software was used to construct a first-order multi-factor cross-correlation model of the four basic variables, and thus the precision of the structural foundation for the subsequent path analysis was ensured. The particular experimental results are as follows: Table 4. At the item observation level, all the factor loadings of the measured latent variables were relatively strong; the standardized loadings of the digital leadership items ranged from 0.753 to 0.897, those for psychological empowerment were from 0.656 to 0.872, those for organizational formalization were between 0.717 and 0.883, and those for employee bootlegging ranged from 0.777 to 0.863. All loadings were above the 0.6 threshold and thus all observed variables were considered suitable indicators of the corresponding latent construct.

**Table 4:** Results of confirmatory factor analysis.

Variable	Item coding	Standardized loadings	AVE	CR
Digital leadership	Q1	0.753	0.672	0.924
	Q2	0.773		
	Q3	0.778		
	Q4	0.836		
	Q5	0.897		
	Q6	0.871		
Psychological empowerment	Q1	0.656	0.679	0.962
	Q2	0.735		
	Q3	0.795		
	Q4	0.825		
	Q5	0.853		
	Q6	0.872		
	Q7	0.851		
	Q8	0.845		
	Q9	0.844		
	Q10	0.856		
	Q11	0.863		
	Q12	0.868		
Organizational formalization	Q1	0.717	0.677	0.936
	Q2	0.776		
	Q3	0.803		
	Q4	0.828		
	Q5	0.869		
	Q6	0.871		
	Q7	0.883		
Employee bootlegging	Q1	0.777	0.66	0.907
	Q2	0.797		
	Q3	0.799		
	Q4	0.825		
	Q5	0.863		

In terms of fit indices, χ^2^/df = 0.942, RMSEA = 0.037, and indicators such as CFI, TLI, and IFI all meet the recommended standards, indicating that the measurement model fits the actual data well.

The Average Variance Extracted (AVE) of all latent constructs is greater than 0.50, and the Composite Reliability (CR) exceeds the upper limit of 0.70; thus, both the measurement system and the internal consistency have met the criteria for good convergent validity.

### 4.6 Discriminant validity and correlation analysis

Discriminant validity aims to confirm statistically significant distinctions among latent variable constructs. To verify the discriminant validity of the items, the original Fornell-Larcker criterion was proposed: the absolute value of the standardized correlation coefficient between any two constructs must be less than the square root of their Average Variance Extracted (AVE). First, the Pearson correlation coefficients of the four main variables were computed in this study, and the results are shown in Table 5. Digital leadership showed a positive correlation with employee bootlegging (*r* = 0.424, *p* < 0.001), as well as a positive relationship with psychological empowerment (*r* = 0.517, *p* < 0.001) and a slight negative correlation with organizational formalization (*r* = −0.031, *p* < 0.001), thus providing a solid basis for the next step of moderated mediation analysis.

**Table 5:** Results of discriminant validity test for each dimension.

Variable	M	SD	1	2	3	4
Digital leadership	4.0	1.644	(0.82)	0.517	−0.031	0.424
Psychological empowerment	4.0	1.652	0.517	(0.824)	0.065	0.417
Organizational formalization	4.0	1.65	−0.031	0.065	(0.823)	0.101
Employee bootlegging	4.0	1.632	0.424	0.417	0.101	(0.812)

The figures in diagonal brackets are the square roots of AVE, and all correlation values are statistically significant at the *p* < 0.01 level.

### 4.7 Model fit test

Model fit in Structural Equation Modelling (SEM) is to determine how well the conceptual system fits the sample covariance matrix empirically. The full framework of fit statistics in organizational psychology will be employed to examine how well the established model fits with empirical data in this study; thus, many metrics, such as the chi-square to degrees of freedom ratio, absolute values, and incremental fit indices, will be used for all-around assessment of the model. The results are presented in Table 6; all of the primary fit indices have reached the desired range, and specifically, the chi-square-to-degrees-of-freedom ratio (CMIN/DF) of 0.942 falls within the desirable range of 1–3; the absolute fit index RMSEA is 0.037 and below the critical value of 0.05; and all incremental fit indices exceed the judgment criterion of 0.9. This shows that the moderated mediation model built in this study fits well with the actual survey data and provides a scientific basis for the following path estimation and hypothesis testing.

**Table 6:** Model fit.

Indicator	CMIN/DF	RMSEA	CFI	TLI	IFI	NFI	RFI
Numerical value	0.942	0.037	0.98	0.97	0.985	0.94	0.92
Standard	1 ~ 3	<0.05	>0.90	>0.90	>0.90	>0.90	>0.90

### 4.8 Path analysis and hypothesis testing

With a good model fit, path coefficient analysis will be used to investigate the connections among the independent variables, mediating variables, moderating variables, and dependent variables in this study. The path coefficients show the strength of the direct influence of one variable on another, and a Critical Ratio (C.R.) is used to determine whether this path has a significant effect. Table 7 shows the specific contents. At the beginning, digital leadership has a positive impact on psychological empowerment (*β* = 0.522, *p* < 0.001), and thus supports Hypothesis H1: that is, more prominent digital leadership is related to a higher level of psychological empowerment for employee. Psychological empowerment is also positively related to the propensity for bootlegging (*β* = 0.269, *p* < 0.001), and thus supports Hypothesis H2: higher levels of psychological empowerment are expected to be associated with higher levels of employee bootlegging. Importantly, the interaction between psychological empowerment and organizational formalization shows a positive effect on employee bootlegging (*β* = 0.127, *p* < 0.01), indicating that organizational formalization acts as a significant positive moderator of psychological empowerment and employee bootlegging; that is, as organizational formalization increases, the positive reinforcement effect of psychological empowerment on the activity of bootlegging gradually intensifies. Therefore, Hypothesis H4 is supported.

**Table 7:** Path coefficients of the structural equation model.

Path	Unstandardized coefficients	S.E.	C.R.	*p*	Significance
Digital Leadership → Psychological Empowerment	0.522	0.044	11.956	<0.001	***
Organizational Formalization → Psychological Empowerment	0.081	0.044	1.866	0.063	ns
Digital Leadership → Employee Bootlegging	0.269	0.05	5.352	<0.001	***
Psychological Empowerment →Employee Bootlegging	0.269	0.05	5.371	<0.001	***
Organizational Formalization → Employee Bootlegging	0.087	0.043	2.027	0.043	*
Psychological Empowerment × Organizational Formalization → Employee Bootlegging	0.127	0.025	5.084	<0.001	***

*** indicates *p* < 0.001, ** indicates *p* < 0.01, * indicates *p* < 0.05, ns indicates not significant.

**Figure 2:**
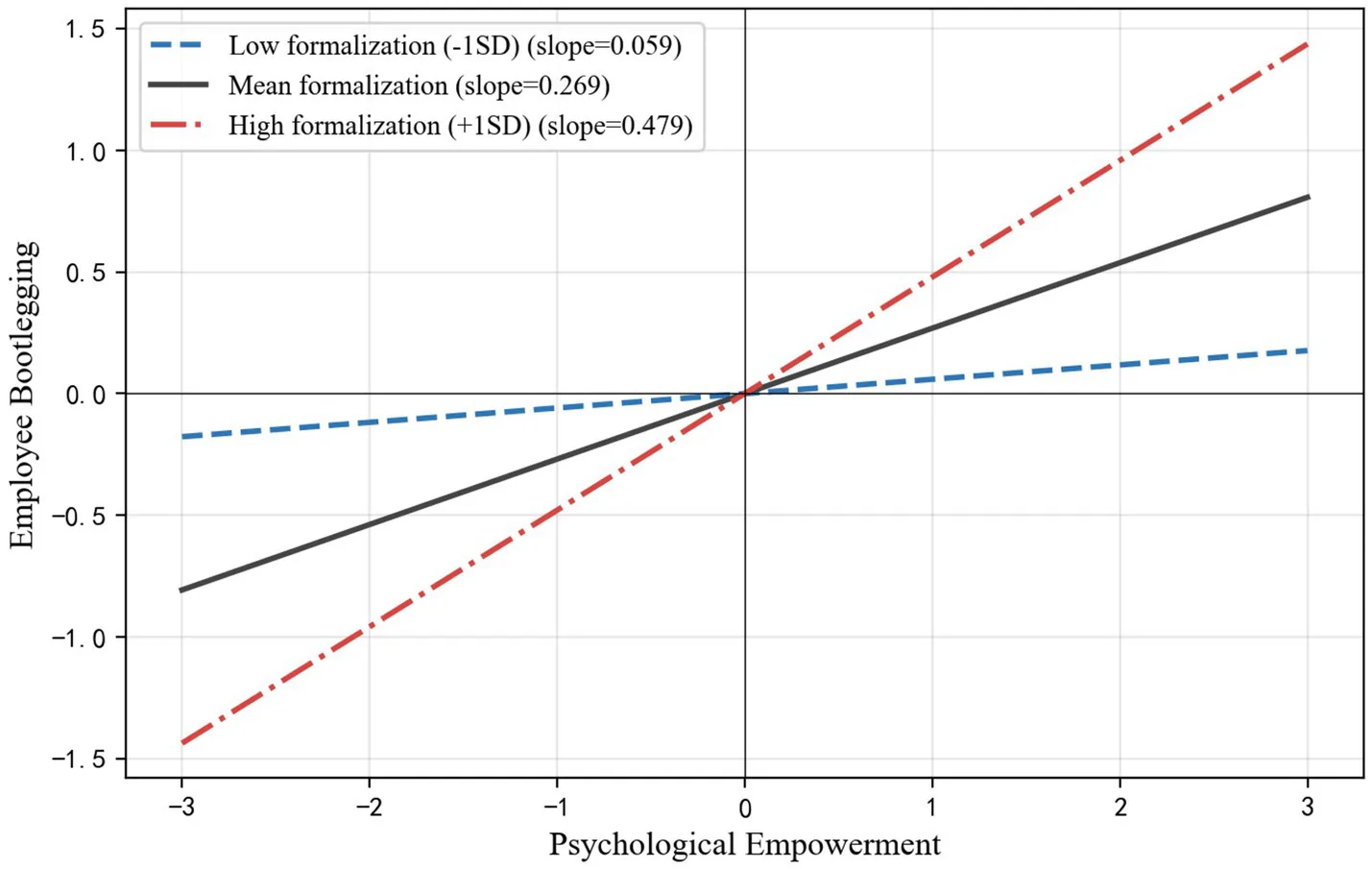
Simple slope moderation effect diagram.

To show more clearly that organization formalization is a moderator, a simple slope analysis was also conducted in this study, and the specific results are presented in Table 8. When organizational formalization was at its lowest (−1SD), the positive effect of psychological empowerment on employee bootlegging was still relatively weak (*β* = 0.059); however, at the highest level of organizational formalization (+1SD), this positive effect increased significantly (*β* = 0.479, *p* < 0.001). Therefore, it can be confirmed that H4 is correct; that is to say, the more formalized the organization, the stronger the motivational impact of psychological empowerment on bootlegging. A simple slope moderation effect diagram is shown in Figure 2.

**Table 8:** Results of simple slope test.

Level of the moderating variable	Simple slope	Standard error	t-value	*p*-value	Significance
Low (−1 SD)	0.059	0.063	0.935	0.3506	ns
Mean	0.269	0.05	5.371	<0.001	***
High (+1 SD)	0.479	0.066	7.21	<0.001	***

### 4.9 Mediation effect and moderated mediation test

Bootstrap resampling technology was employed, as proposed by Preacher and Hayes, to examine the stability of the path coefficients further and to assess the overall extent of mediation and moderated mediation in the system. A total of 5,000 resampling iterations have been set. If the 95%-confidence interval of a particular path does not include 0, then that path is considered statistically significant. Initially, at the mean level of the moderating factor, the mediation role of psychological empowerment in the mediation path of digital leadership and employee bootlegging was 0.141; with a Bootstrap standard error of 0.0296 and a 95% confidence interval of [0.086, 0.201], it did not include 0 and thus indicated that the effect of mediation by psychological empowerment was significant, thus supporting Hypothesis H3. Subsequent examination of the moderated mediation effect (H5) was conducted, and the results of the conditional indirect effect analysis are presented in the table below. When organizational formalization is low (-1SD), the indirect effect is 0.031 (95% CI = [−0.041, 0.103]); at the other end, when organizational formalization is high (+1SD), the indirect effect is relatively larger at 0.25 (95% CI = [0.181, 0.326]). This indirect effect systematically strengthens with an increase in the degree of organization, and it remains statistically significant at all levels of organizational formalization. Hayes (2015) found that the final Index of Moderated Mediation was 0.0665, with a Bootstrap standard error of 0.0134 and a 95% confidence interval of [0.0422, 0.0942]; since this range does not include 0, it can be concluded that moderated mediation has been achieved. Based on the above results, the positive moderating effect of organizational formalization on the mediation path of digital leadership → psychological empowerment → employee bootlegging is supported, thus validating Hypothesis H5. The details are shown in Table 9.

**Table 9:** Conditional indirect effects and index of moderated mediation (bootstrap = 5,000).

Level of the moderating variable	Indirect effect	Boot SE	95% CI lower	95% CI upper
Low (−1 SD)	0.031	0.0367	−0.041	0.103
Mean	0.141	0.0296	0.086	0.201
High (+1 SD)	0.25	0.0371	0.181	0.326
Index of moderated mediation	0.0665	0.0134	0.0422	0.0942

The moderated mediation index is the INDEX indicator proposed by Hayes (2015), where a CI that does not include 0 indicates a significant moderated mediation effect.

## 5 Conclusion and discussion

### 5.1 Research conclusion

The first one is that Self-Determination Theory (SDT) will be used for this study. Through strict structural equation modelling (SEM) and bootstrap testing on 387 valid sample data points, systematically explore how organizational environments in the digital age affect employees’ informal innovative behavior. The four main conclusions are as follows.

First, digital leadership is a key environmental antecedent that stimulates employees’ psychological empowerment. According to the above experiments, digital leadership has promoted psychological empowerment. Therefore, given that digitalized society is changing rapidly and profoundly at present, if leaders are trained in new technologies and provide support for using these new technologies, along with actively introducing flexible working patterns that can quickly adjust to new technological systems, then the uncertainty employees feel in times of change will be reduced. This kind of leadership provides resource support and is willing to accept trial-and-error; it meets employees’ needs for competence and autonomy, allowing them to feel a high degree of work control, self-efficacy and a strong sense of purpose in their work at the psychological level.

Second, psychological empowerment also provides a medium for the impact of digital leadership on employee bootlegging. Therefore, based on the results of the above study, positive digital leadership does not automatically result in bootlegging; instead, it needs to be promoted organically at the organizational level by inspiring the staff. Psychological empowerment is another reason for this wish. When employees are strongly psychologically empowered by digital enablement, they no longer wish to mechanically achieve routine KPIs. Instead, they spontaneously develop the desire to go beyond their own work and drive technological innovation in the company. This strong inner drive provides them with the psychological capital to take the risks of deviation, such as working without permission or using time for personal purposes.

Third, as an institutional constraint, organizational formalization is conducive to supporting the conversion of psychological empowerment into bootlegging. It is not that there is no problem, however. Based on the above data, it is clear that organizational formalization has an enhancing effect; that is to say, in a highly formalized environment with stringent rules and strict approval chains, the impetus for psychological empowerment to drive the behavior of bootlegging is relatively stronger compared to less formalized, more flexible organizations. This means that when highly empowered employees encounter rigid bureaucratic barriers, their innovative enthusiasm is not extinguished but rather generates a squeezing effect. To protect fleeting innovative inspiration, they have no choice but to bypass red tape and channel their innovative exploration into covert informal channels.

Fourth, within the organization, a moderated mediating transmission chain based on “environment-motivation-institution-behavior” has been formed. The study confirms that the degree of organizational formalization not only moderates the latter part of the pathway but also positively moderates the entire mediating chain. Overall, this shows that the actual results of digital leadership have been limited by the entire macro-institutional environment. In a rigid system, the freedom and power granted to employees by digital leaders end up taking the form of “underground deviance” that the organization cannot fully control at the top.

### 5.2 Theoretical contribution

This study has discussed the relevant research on organizational behavior and innovation management, and the main theoretical contributions are as follows.

First, it expands the scope of influence for digital leadership and no longer assumes that good leadership will result in obedience. Many of the recent studies on digital leadership have investigated how it is positively correlated with employees’ formal task performance, compliant creativity, or subjective well-being (Cortellazzo et al., 2019; Zeike et al., 2019). This study innovatively links it to seemingly destructive bootlegging behavior, revealing the potential spillover effects or even unintended consequences of digital empowerment. This provides a more tension-filled and dialectical theoretical perspective for academia to understand the effectiveness of leadership in the digital era, confirming that positive antecedents can also induce gray behaviors with hidden risks. This finding engages in a theoretical dialogue with the study by Zhang et al. (2026b), which revealed the cognitive pathway through which digital leadership stimulates employees’ improvisational ability via work flow. In contrast, the mediating role of psychological empowerment identified in this study constitutes a motivational pathway. Although the mechanisms of these two pathways differ, they both point to the fact that digital leadership can activate employees’ action tendencies beyond conventional role norms. At the same time, this study advances Self-Determination Theory (SDT). The reason digital leadership can effectively activate psychological empowerment is that it addresses employees’ unique competency anxiety and desire for autonomy in the digital context. This suggests that the effect of “environmental support” on fulfilling psychological needs is not generalized but highly dependent on the actual psychological state of employees in a specific era.

Second, it deepens the research on the antecedent mechanisms of bootlegging, establishing a legitimate explanation of positive intrinsic drive based on Self-Determination Theory (SDT). Traditional management often views deviant behavior as a violation and disruption of organizational discipline. However, by introducing psychological empowerment as a core internal motivational variable, this study confirms that employees engage in underground activities not out of self-interested malicious sabotage, but due to their highly activated sense of work meaning and competence (Criscuolo et al., 2014; Mainemelis, 2010). This paper expands the application range of SDT to abnormal organizational behavior and presents research that finds that bootlegging is, in fact, a type of low-level expression of intrinsic motivation in the face of real-life barriers. This conclusion contrasts with the findings of Zhang et al. (2026a) on responsible leadership driving sustainable performance through knowledge search, where the former represents a “cognition-driven” pathway more likely to lead to behaviors aligned with organizational expectations. In contrast, the psychological empowerment revealed in this study represents a “motivation-driven” pathway, more likely to spill over into informal behavioral boundaries when institutional constraints are present. Furthermore, integrating the findings of Sun et al. (2024) on transformational leadership promoting deviant innovation through creative self-efficacy, and Liu et al. (2025) on authentic leadership promoting bootlegging through affective commitment, it can be seen that multiple forms of positive leadership converge on the common endpoint of bootlegging through distinct mediating mechanisms. From the perspective of SDT, bootlegging can be understood as “the redirection of intrinsic motivation when institutional constraints are encountered.” SDT traditionally links intrinsic motivation to positive, compliant behavioral outputs, but this study suggests that when the institutional environment limits the feasible space for positive behaviors, intrinsic motivation does not dissipate but instead shifts to informal channels for release. The relationship between motivation and behavior is not unidirectional; the shaping of the behavioral option set by the institutional environment is equally significant.

Thirdly, it shows the contradictory role of organizational formalization in the innovation process and adjusts the boundary effects of institutional contexts. Early studies generally believed that bureaucratic formal institutions mechanically suppressed the motivation for individual innovation and thus served as a natural obstacle to innovation (Adler and Borys, 1996). Based on this study, there is no longer a one-way link; employees who have achieved high psychological empowerment through digital leadership are no longer merely recipients but also activators of highly formalized institutions (Globocnik and Salomo, 2015). Instead of lowering the incentive for innovation, institutions have now been changed less formally. Expand the theoretical foundation for the study of the dual characteristics of organizational formalization and provide empirical evidence on how control and autonomy affect organizations. This counterintuitive finding corroborates the paradox identified by Zhao et al. (2025). That paradox is that organizations encourage autonomy while imposing constraints through formal rules. This combination thereby fosters bootlegging innovation. Our finding also corroborates the argument by Wang Q. et al. (2025). They argue that “creativity expectations, combined with blocked formal channels, compel employees to break rules.” Taken together, organizational formalization plays a role of “catalytic friction.” It does not weaken innovation motivation itself. Instead, it redirects the channels through which motivation is expressed. Furthermore, we integrate this study with Zhang et al. (2026a,b) findings on the moderating role of psychological safety. Psychological safety facilitates the transformation of positive psychological states into constructive behaviors. However, organizational formalization diverts the same psychological states toward unconventional channels when obstacles arise. This finding also refines SDT’s understanding of “contextual constraints.” Traditional SDT research typically views contextual constraints as factors that undermine psychological needs and reduce motivation. This study, however, demonstrates that constraints can influence behavior through different pathways. Highly formalized systems do not diminish the innovation motivation of employees with high psychological empowerment. Otherwise, the effect of psychological empowerment on bootlegging would not be amplified under high formalization. Instead, formalization blocks official innovation channels. This forces motivation to find expression through informal avenues. This suggests that contextual constraints can either weaken motivation or alter the direction of behavior. This allows SDT to offer a more nuanced explanation of the interplay between institutional environments and individual motivation.

### 5.3 Practical insights

Given the innovation anxiety and management problems that have occurred in the course of digitalization, this paper offers the following practical recommendations for organizational design and human resources in enterprises.

First, change the form of the digital management model from tool empowerment to deep psychological empowerment. Although the construction of a “hard environment” with AI software and cloud computing platforms is being carried out, the establishment of a digital leadership model by the management should also be strengthened at the same time (Erhan et al., 2022). Managers should be aware that in real terms, the first change is in the minds of the people. Distribute the power to decide and build a fault-tolerant structure to make the staff feel more capable and independent in using the technology. Psychological empowerment can be received from outside, but it is only then that the organization will generate an internal will to change among its staff.

Second, acknowledge and guide bootlegging by establishing an innovation sandbox mechanism that combines both restriction and facilitation. Research indicates that in the digital era, bootlegging has a strong inherent inevitability. Therefore, managers should not adopt a rigidly defensive or severely punitive attitude, treating “underground innovation” as a menace (Augsdorfer, 2005). Instead, companies should recognize the early incubation value of informal exploration and explore the establishment of inclusive “innovation sandboxes” or internal maker spaces. For example, by setting up small-scale informal exploration funds or allowing a certain proportion of discretionary time, a buffer zone for partial legitimization of covert innovation can be created, thereby bringing high-value bootlegging into the organization’s formal purview early on and reducing the risk of strategic loss of control.

Third, to overcome the “big company disease,” enterprises must simultaneously implement de-bureaucratization while advancing digital transformation. The data from this study sounds an alarm: rigid bureaucratic systems are the primary culprit forcing outstanding employees to engage in “underground operations.” If companies only empower digitalization on the business side while retaining lengthy and rigid approval processes on the management side, employees’ high enthusiasm will only clash violently with the system (Nambisan et al., 2017). Therefore, enterprises must simultaneously simplify internal project initiation and resource approval processes, reduce the absolute formalization of the system, and ensure that bottom-up innovative ideas have smooth formal channels for expression and incubation, thereby achieving the optimal resonance between individual innovation motivation and the organization’s overall strategy. The findings of this study also suggest to managers that the high incidence of deviant innovation may not necessarily result from employees’ weak sense of discipline, but rather reflects blockages in the organization’s formal innovation channels. Managers should view the distribution of bootlegging as a feedback signal of institutional design, focusing on unblocking rather than simply blocking informal innovation.

### 5.4 Limitations and prospects

Although this study has made some contributions to the development of theory and provided some empirical data, it is not perfect and has certain shortcomings that need to be corrected in the future. First, Cross-sectional questionnaires were used to collect data at the beginning of this study. Although it has good logical foundations, it is still difficult to determine whether the factors were active at the same time. Further research will be carried out in the form of a long-term follow-up or a context experiment for confirmation. Second, as a covert and sensitive informal behavior, bootlegging relies entirely on self-reports from employees, which may not completely eliminate the interference of social desirability bias. Subsequent studies could attempt to incorporate supervisor evaluations, peer assessments, or diary studies to enhance the objectivity of behavioral measurement. Finally, the sample of this study covers multiple types of enterprises. While this ensures the generalizability of the conclusions, it may obscure the impact of industry heterogeneity, such as differences in tolerance for deviant behavior between high-tech industries and traditional manufacturing. Future research could focus on specific industry characteristics or introduce macro-contextual variables such as organizational innovation climate and cultural power distance to delve into the differential boundary conditions of the antecedent mechanisms of bootlegging across different cultural and industrial contexts.

## Data Availability

The original contributions presented in the study are included in the article/supplementary material, further inquiries can be directed to the corresponding author.
